# Efficiency of a solar collector system for the public building depending on its location

**DOI:** 10.1007/s11356-019-05077-2

**Published:** 2019-04-18

**Authors:** Dorota Anna Krawczyk, Mirosław Żukowski, Antonio Rodero

**Affiliations:** 1 Department of HVAC Engineering, Bialystok University of Technology, Bialystok, Poland; 2 School of Engineering Sciences of Belmez, University of Cordoba, Cordoba, Spain

**Keywords:** Solar collectors; Renewable energy; DHW, Efficiency, Beam radiation, Solar system, Simulation

## Abstract

Due to a technologic progress, a growth in the renewable energy markets including a high number of manufactures coming to being, the Renewable Energy Sources (RES) are both a tool for mitigating climate changes and investments that can provide direct economic profits and reduce a political or economic dependence resulting from import of fossil fuels. One of the many key solutions toward fulfilling the global increasing demand for energy and reduction of CO_2_ emissions is applying solar technologies. This paper presents the results of the analysis conducted for a small public office building located in Bialystok (Poland), where solar collectors were considered as the RES for domestic hot water (DHW) system, on the understanding that existing oil boiler would be an additional source. Low values of recommended water consumption in office buildings resulted in a low energy demand. However, concerning a potential of all office governmental buildings in Poland, it would be possible to reduce CO_2_ emission by 17,248 tonnes. In the comprehensive analysis, the same building in two more locations (Cordoba (Spain) and Kaunas (Lithuania)) was considered using simulation tools delivered within the framework of VIPSKILLS project as well as EnegyPlus software. The results allow to compare the mean monthly efficiency of systems or number of collectors necessary to deliver similar amount of solar energy.

## Introduction

Global energy demand has been increasing due to a growing urban population; however, it is worthy to highlight that improved energy access is a crucial means of advancing the quality of life and socioeconomic status of a country’s growing population that could improve the citizens’ contribution to economic growth and environmental sustainability (Elum et al. 2017). Renewable energy sources (RES) play a key role in a sustainable economic growth and help to meet the increasing energy demand. Moreover, they conduce to a reduction of CO_2_ emissions that are primarily generated through the consumption of fossil fuels. Usage of renewable and non-renewable energy sources in the context of greenhouse gas (GHG) emission was discussed by Zaidi et al. (2018), Mensah et al. (2018), or Azad et al. (2015). Recently, the situation of Renewable Energy Sources has shifted considerably and their large-scale deployment can be observed. Most countries pay attention at applying the RES in new investments and gradual elimination of existing conventional sources, whereas part of countries even considers the possibility of achieving a share of 100% of national energy consumption from the RES.

Solar energy is considered as abundant, widely distributed, and pollution-free that makes it a highly sought-after kind of energy in a great deal of fields. A solar collector market in many countries all over the world has had its prime time lately. As noted by Qi and Zhang (2017), solar technology provides clean renewable energy; does not cause land, environmental, and ecological problems; and conforms to the concept of sustainable development unlike traditional power generated by coal-fired power technology. There are variety of factors, including a local solar radiation, a technology progress, costs, local manufactures’ engagement, an economic government support, or an environmentally consciousness raising relevant to a mitigating climate change, that influence local markets’ development (Krawczyk 2019, Krawczyk et al. 2018). The thermal energy consumption in development countries has been rising significantly since 2000 (Paramati et al. 2018; ESTIF 2016). According to the Directive (2009), the EU countries target is to produce at least 20% of all energy from renewable energy sources by 2020. One of the possibilities is an application of solar thermal DHW (domestic hot water) systems in both new design buildings and in lieu of conventional energy sources in existing ones. Implementation of the solar thermal systems provides numerous opportunities and benefits, including greenhouse gas emission reductions, energy security, improved energy access, grid stability and resilience, improved quality of life, and new economic development opportunities. It can also mitigate burdens on local governments and infrastructure by reducing pressure on the national power system and diminishing pollution produced by conventional energy sources (UNEP 2014; Krawczyk et al. 2018). The results of the analysis of the efficiency of systems with plate and tube solar collectors in residential buildings in Poland and Spain were presented by Krawczyk et al. (2019).

This study is a continuation of a preliminary research presented by Krawczyk et al. (2018) that was focused on the optimal tilt angle for solar collectors in different locations. The aim of this paper is to compare the purposefulness of solar collector installation in small offices in selected countries. Comparison was conducted for towns located in Poland, Lithuania, and Spain, to show the differences in efficiency of systems, amount of energy gathered from solar installations, etc.

## Materials and methods

**Fig. 1:**
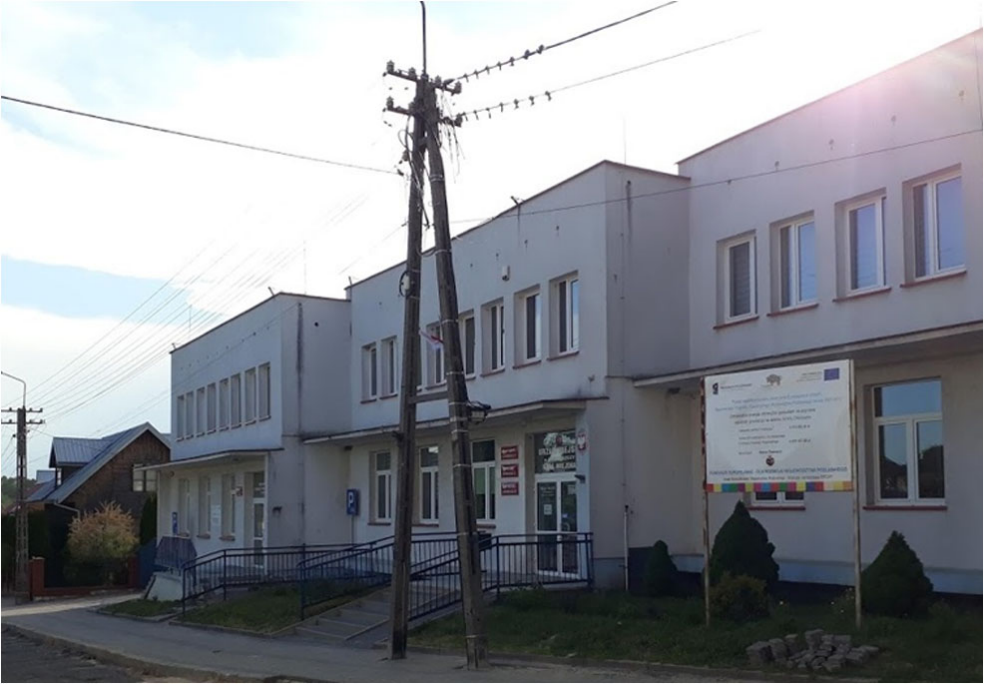
Analyzed office building (photo by D.A.Krawczyk)

The analysis for the building employed as the Municipal Office in a town located in the northeast part of Poland (Fig. 1) was conducted. This building was selected, as a representative of local government buildings, to generalize obtained results for the others.

**Fig. 2:**
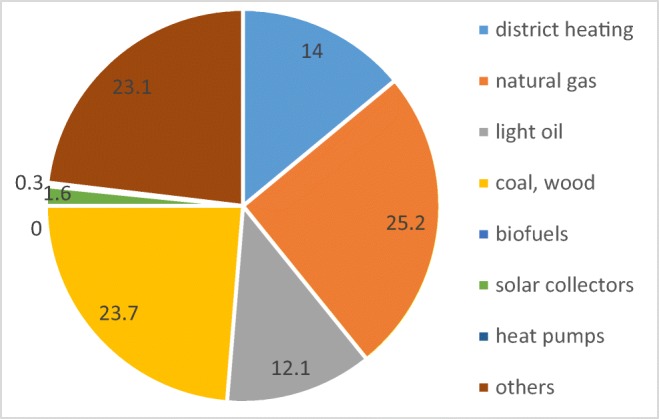
Share of energy sources for DHW in governmental buildings in Poland (source: own elaboration based on CSES GUS 2015)

As shown in Fig. 2, most local government buildings built before 2013 used mostly fossil fuels and heat from local power plants (in most cases also utilizing fossil fuels) and natural gas. As noted in Report (CBES GUS 2015) last year, about 7% of DHW systems in local government offices were modernized. Most coal power plants were changed to natural gas ones. Part of existing natural gas local plants was improved by applying solar collectors, while other buildings started to be supplied from a district heating network. Annual energy consumption in local government buildings was estimated as 264.8 TJ.

The analysis was conducted for a domestic hot water system, used by 54 employees, with water intakes in toilets and social rooms, in Poland. Moreover, two additional locations of the building (Cordoba (Spain) and Kaunas (Lithuania)) were considered, to compare the economic and ecological effect coming from the modernization of the heat source and applying solar collectors in different countries.

For about 15 years, an oil boiler has been used as a heat source for HVAC system and partly for DHW installation, whereas a few intake points had their own electrical heaters. This paper discusses possibility to apply solar collectors for DHW system in the building.

### Hot water consumption

Hot water consumption in the office building was estimated using the national recommendations:

- 3 dm^3^/day person in Spain (IDAE 2009),
- 7 dm^3^/day per person in Poland (Regulation 2015),
- 5–7 dm^3^/day per person in Lithuania (RSN 26-90 1991).

The monthly energy consumption for DHW preparation Q_DHW_ was estimated from Eq. (1):

1 $$ {Q}_{\mathrm{DHW}}(GJ)={C}_{\mathrm{e}}\ {D}_i\rho\ \left({T_{\mathrm{H}}}^i-{T_{\mathrm{C}}}^i\right) $$

where *C*_e_ is the specific heat capacity of water in J/kgK, *ρ* is the density of water in kg/m^3^, *D*_*i*_ is the hot water demand in *i* month in m^3^/month, *T*_H_^*i*^ is the storage temperature of hot water in °C, and *T*_C_^*i*^ is the temperature of network cold water in *i* month in °C.

The value of *T*_C_ depends on outdoor air temperature and is a function of the average annual outdoor air temperature *T*_AMB,A_ and maximum difference in monthly average outdoor air temperature ⍙*T*_AMB,D_; wherefore, temperature of network cold water was calculated using Eq. (2). The method for estimating the main water temperature was developed by Hendron et al. (2004) and Burch and Christensen (2007).

2 $$ {T}_{\mathrm{C}}=\left({T}_{\mathrm{AMB},\mathrm{A}}+6\right)+\mathrm{ratio}\left(\frac{\varDelta_{\mathrm{AMB},\mathrm{D}}}{2}\right)\sin \left[0.986\left(\mathrm{day}-15-\mathrm{lag}\right)-90\right] $$

where:

$$ \mathrm{ratio}=0.4+0.01\left({T}_{\mathrm{AMB},\mathrm{A}}-44\right); $$

$$ \mathrm{lag}=35-\left({T}_{\mathrm{AMB},\mathrm{A}}-44\right). $$

with *T*_AMB,A_ and Δ*T*_AMB,D_ in °F.

**Fig. 3:**
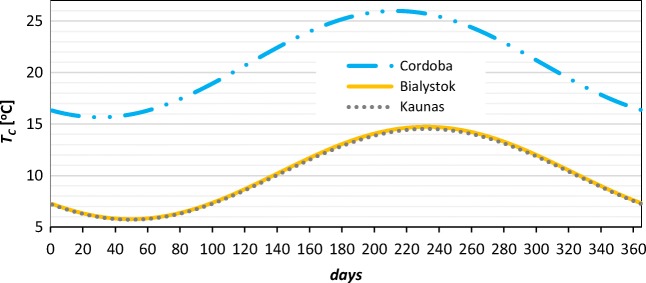
Changes of network water temperature during a year in Cordoba, Bialystok, and Kaunas (source: own elaboration)

Annual profile of network cold water was presented in Fig. 3.

The useful heat gain from solar collector was calculated based on Eq. (3):

3 $$ {q}_{\mathrm{SOL}}(W)=\eta {I}_{\mathrm{SOL}}{A}_{\mathrm{g}}, $$

where *η* is an efficiency of a collector, *I*_SOL_ is the total incident solar radiation in W/m^2^, and *A* is gross area of the collectors in m^2^.

Equation (4) was used to estimate a thermal efficiency of the collectors.

4 $$ \eta ={c}_0+{c}_1\left({T}_{\mathrm{in}}-{T}_{\mathrm{a}}\right)/{I}_{\mathrm{SOL}}+{c}_2{\left({T}_{\mathrm{in}}-{T}_{\mathrm{a}}\right)}^2/{I}_{\mathrm{SOL}} $$

where *c*_0_ is a zero-loss collector efficiency, *c*_1_ is a heat loss coefficient in Wm^−2^ K^−1^, *c*_2_ is the temperature dependence of the heat loss coefficient in Wm^−2^ K^−2^, *T*_in_ is the collector inlet temperature in K, and *T*_a_ is the ambient air temperature in K.

The energy gathered from the solar domestic hot water system within a month was calculated using Eq. (5).

5 $$ {Q}_{\mathrm{SOL}}\left(\mathrm{kWh}\right)=n\cdot 24\cdot {q}_{\mathrm{SOL}}\cdot {10}^{-3}, $$

where *n* is number of days in each month.

### Description of the analyzed system with solar collectors

The system with the plate water collector with a gross area 2.05 m^2^ was analyzed. Main parameters of panels are shown in Table 1. In fact, the coefficients shown in the table have a positive value, but they should be substituted to Eq. (4) with a minus sign; thus, negative values were presented.

**Table 1:** Basic parameters of solar collectors (source: own elaboration)

Zero-loss collector efficiency [−]	Heat loss coefficient [Wm^2^K^−1^]	Temperature dependence of the heat loss coefficient [Wm^2^K^−2^]	Incident angle modifier coefficients b_0_ and b_1_	Absorption coefficient	Sheeting glass [m]
0.784	− 3.64	− 0.00185	− 0.121 and 0.0	0.95	0.003

**Fig. 4:**
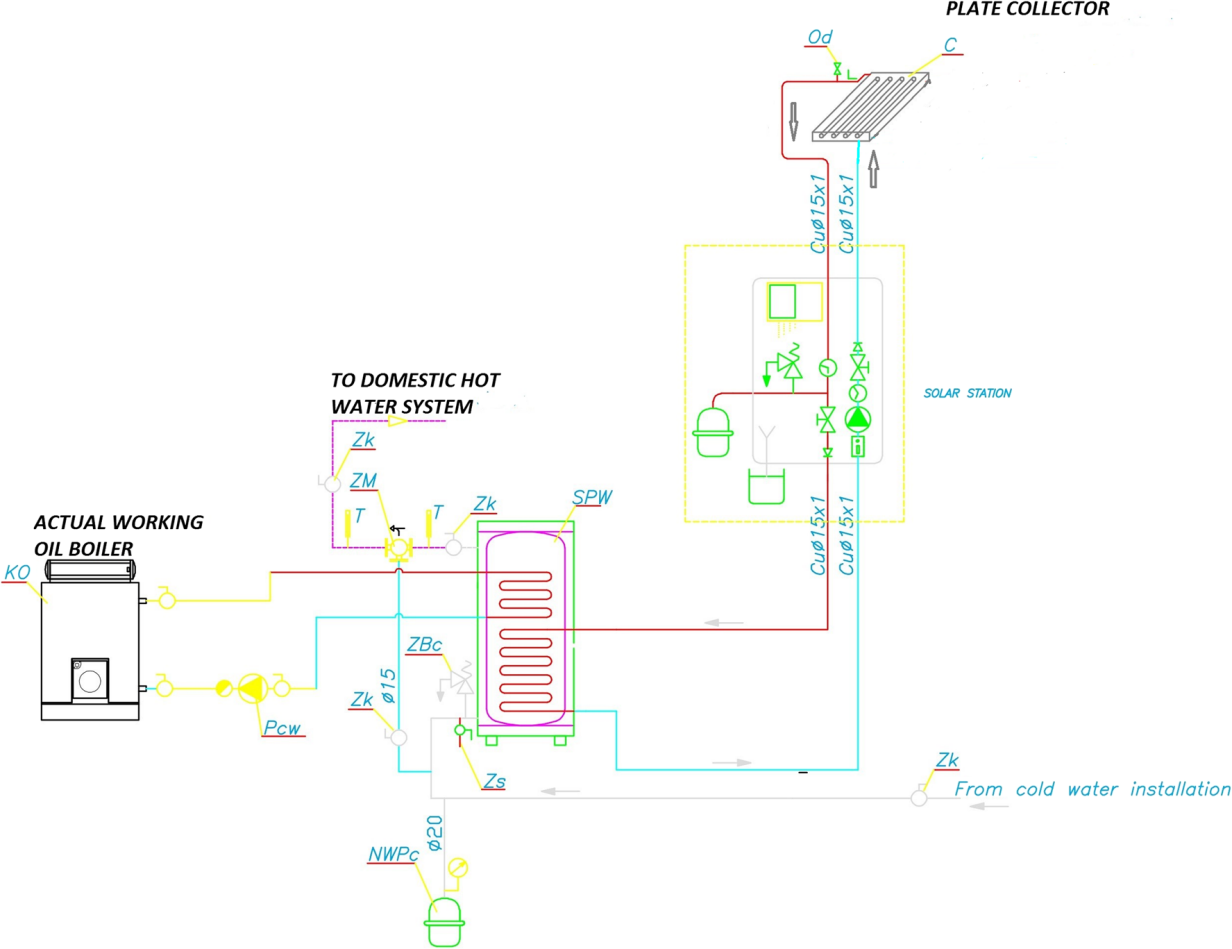
Scheme of the solar collector system: P_cw_, pump; NWP_c_, expansion vessel; C, collector; KO, boiler; SPW, water tank; Z_k_, cut-off valve; Z_s_, blow-off valve; ZB_c_, safety valve; ZM, control valve; Od, air valve; T, thermometer (source: own elaboration)

As shown in Fig. 4, the solar collector supplies the tank (SPW) and is predicted to be a main energy source for the DHW system in summer, whereas the existing oil boiler would work also for the hot water demand in conditions with too low heat gains from the solar source.

**Fig. 5:**
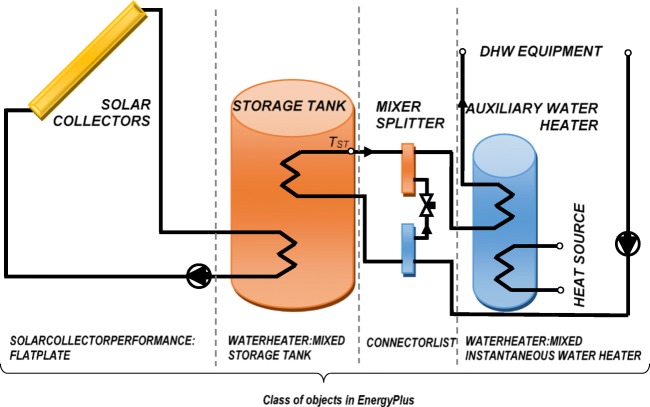
Solar heating system connection diagram set in calculations (source: own elaboration)

The analysis was conducted using EnergyPlus software. In order to estimate energy possible to gather from solar system, it was necessary to build a schema with two-stage water heating (Fig. 5); hence, the DHW installation is composed of the flat plate solar collector, a storage tank, and an auxiliary water heater. In this instance, the storage tank accumulates heat from the solar collector, while the auxiliary water heater provides additional heat if the storage tank water temperature is too low. In this case, it was possible to analyze a variation of temperature of water leaving the storage tank (*T*_ST_) during the whole year.

In simulations, temperature in the water heater was compared with the temperature in the solar collector loop, so the pump was turned on when there were any useful heat gains. The temperature difference on limit (TDonL) was set to 10 °C and the temperature difference off limit (TDoffL) was set to 2 °C. If the temperature difference between collector outlet and storage tank source outlet was above TDonL, the system was turned on, whereas the system was turned off when the temperature difference was below TDoffL again. According to our assumption, the temperature of DHW in the storage tank was set to 60 °C and a mixed temperature at the water tap was set to 50 °C. According to national rules, DHW systems should ensure obtaining at tap points temperature not lower than 50–55 °C, and not higher than 60 °C (WT 2017; Technical Guide 2010). As shown by Ocipova et al. (2012) and Vieira et al. (2018), it is necessary to predict any kind of bacteriolytic protection, whereas Amara et al. (2017) noted that only thermal treatment could completely eliminate Legionella, which is killed almost instantly at 70 °C. Also, national regulations (WT 2017; Technical Guide 2010) recommend a thermal periodic disinfection in 70–80 °C temperature. Thus, an increase of temperature was predicted by delivering of energy by the additional source. However, thermal disinfection is only a short-term activity, so in this analyses, the amount of energy necessary to increase the temperature was not taken into account.

### Description of solar radiation in analyzed locations

**Fig. 6:**
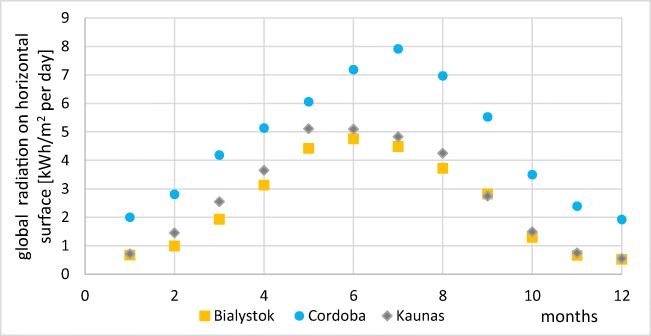
Global monthly radiation on horizontal area in Bialystok, Cordoba, and Kaunas (source: own elaboration)

Main data relevant to solar potential in Bialystok, Cordoba, and Kaunas were presented in Table 2 and Fig. 6. The highest daily global radiation on horizontal area was found in Cordoba in July (4.92 kWh/m^2^day) while the lowest one in Bialystok in December (0.52 kWh/m^2^day). The values of solar radiation in Poland and Lithuania were very similar between November and January, whereas in the rest of year, radiation in Kaunas was found 6–31% higher than in Bialystok. Monthly solar radiation in Spain was during all year much higher than in Poland and Lithuania, reaching its maximum in July.

**Table 2:** Basic parameters of Bialystok, Cordoba, and Kaunas (source: own elaboration)

Parameter	Białystok	Córdoba	Kaunas
Geographic localization	53°07′ North latitude	37°53′ North latitude	54°53′50″ North latitude
	23°10′ East longitude	4°46′ West longitude	23°53′10″ East longitude
Insulation time (h/year)	1780	2900	1670
The minimum daily global radiation on horizontal area kWh/(m^2^day)	0.52 (December)	2.00 (January)	0.55 (December)
The maximum daily global radiation on horizontal area kWh/(m^2^day)	4.76 (June)	7.92 (July)	5.11 (May)

## Results and discussion

### Energy consumption

Theoretical hot water and energy consumption in the analyzed building for all locations was estimated in a range from 162 dm^3^/day in Spain to 378 dm^3^/day in Poland and Lithuania, so the obtain results indicate the solar collector system similar to family houses (Krawczyk et al. 2018.

**Fig. 7:**
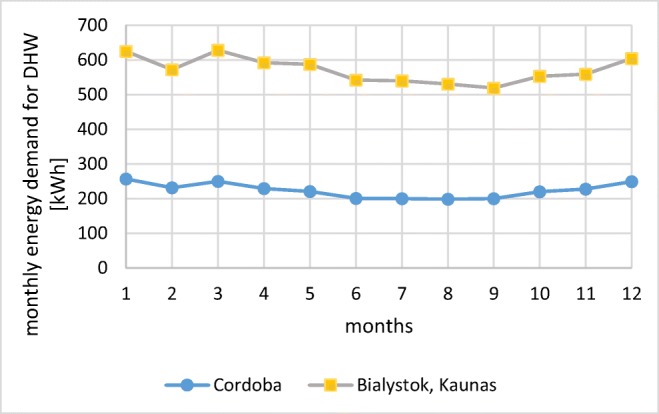
Monthly energy demand for DHW in Bialystok, Cordoba, and Kaunas (source: own elaboration)

Changes in a monthly energy demand, calculated using formula (1), were presented in Fig. 7. Values in Lithuania and Poland are nearly the same because of very similar network water temperature and water consumption per person. Energy demand for DHW in Cordoba was found 2.9–3.5 times lower, as a result of lower water consumption per person recommended by the national regulation (IDEA 2009) and significantly higher temperature of network water (Fig. 3).

### Characteristic of solar system’s work

Firstly, using EnergyPlus software, we simulated the work of system with one solar collector for all cites. In Cordoba, we assumed solar collector installation with a tilt angle of 45°, whereas in Bialystok and Kaunas − 35° to obtain maximum efficiency, as recommended by Krawczyk et al. (2018).

**Fig. 8:**
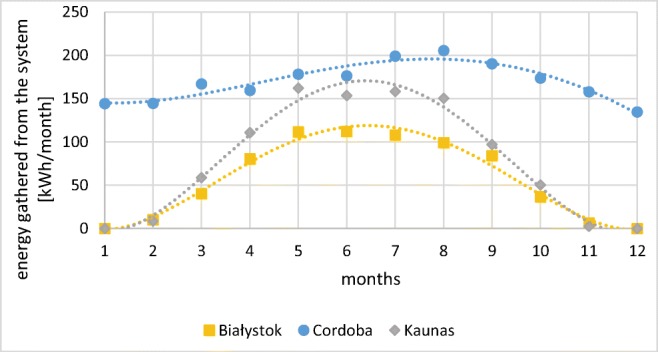
Variation of energy gathered from the system with one solar collector (source: own elaboration)

Energy transfer from the system in Cordoba was found three times higher than in Bialystok (respectively 990.5 kWh/m^2^ of collector and 336.2 kWh/m^2^ of collector), while in Kaunas, obtained value was 464.5 kWh/m^2^ of collector, so 38% higher than in Poland. The monthly variation of energy gains is presented in Fig. 8.

In Spain, monthly gains were the lowest in December (134.51 kWh/month) and the highest in August (205.56 kWh/month). In both Poland and Lithuania in January and December, heat transfer energy was null. The highest values were found in Poland in June (112.17 kWh/month), while in Lithuania in May (160.10 kWh/month).

**Fig. 9:**
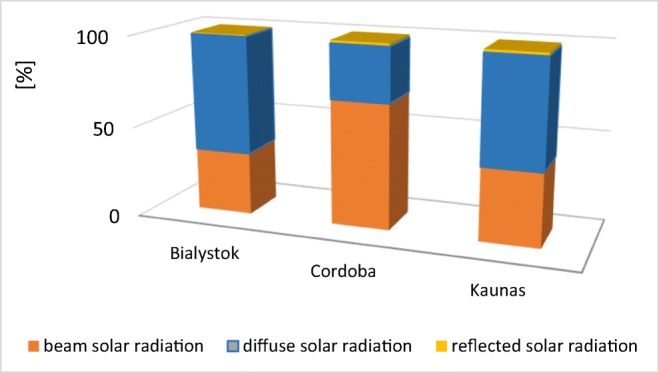
Contribution of beam and diffuse radiation in a total balance (source: own elaboration based on Krawczyk et al. 2018

As demonstrated by Krawczyk et al. (2018), the results of the simulations made using VIPSKILLS (2018) tools showed that a contribution of beam and diffuse radiation for analyzed locations differs significantly. In Cordoba, the beam solar radiation share in the total radiation balance was nearly twice higher than in Bialystok or Kaunas (Fig. 9).

**Fig. 10:**
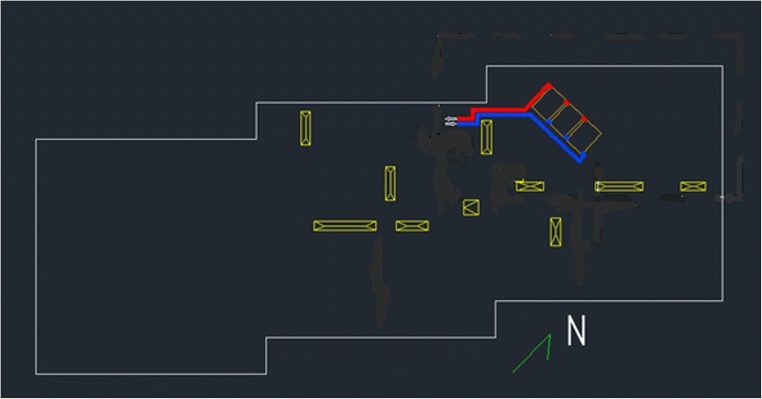
Location of collectors on the roof (own elaboration)

Since the system with one collector was found to be useful only in Spanish case, systems with a number of panels necessary give similar energy gains as in Spain, so two panels in Lithuania and three collectors in Poland were considered (Fig. 10). As the building roof’s area is significantly higher than collectors’ area, it was possible to plan their location avoiding the shielding by different technical elements of the building construction that could decrease the annual heat gain of collectors (Sikula et al. 2013; Żukowski and Radzajewska 2016; Kolendo and Krawczyk, 2018).

**Fig. 11:**
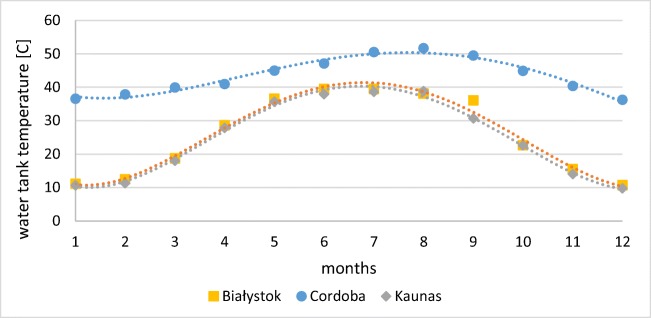
Variation of mean water tank temperature (source: own elaboration)

The temperature in a water tank was simulated, taking into account the temperature and flow of water from solar collector system, the 24-hour tapping pattern, and the supply of the tank by cold network water. The significant aspect of the water storage tank was also energy consumption due to its standing losses. Figure 11 presents monthly variation of water tank temperature. This average annual temperature of water leaving the tank was estimated as 24.8–25.9 in Lithuania and Poland, respectively, whereas in Spain, the value was found as 43.4 °C.

Maximum temperature was found in Cordoba (51.58 °C), whereas minimum temperature for this destination was 36.25 °C. Maximal monthly values obtain in Bialystok and Kaunas were 39.7 °C and 39.9 °C respectively. The lowest temperature of water in a tank (9.78 °C) was estimated for installation in Kaunas in December; however, in Bialystok, the temperature was only slightly higher (10.72 °C). In Poland, the temperature was higher than Spanish minimum value for over 5 months, in turn in Lithuania—only in 3 months.

**Fig. 12:**
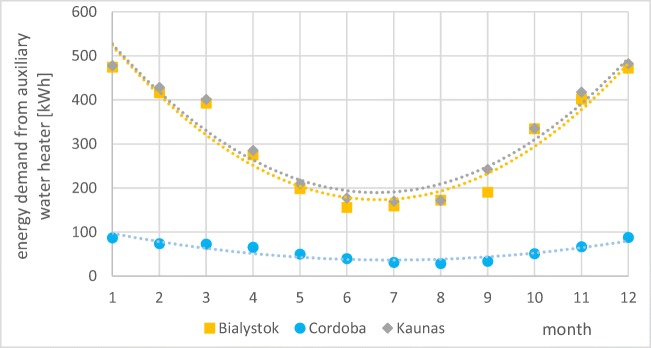
Variation of energy demand gathered from auxiliary water tank (source: own elaboration)

Therefore, to maintain warm water temperature at the recommended level, it is necessary to use additional energy source. Figure 12 shows requisite energy demand from the auxiliary water heater allowing to reach the minimum water temperature at the taps.

**Fig. 13:**
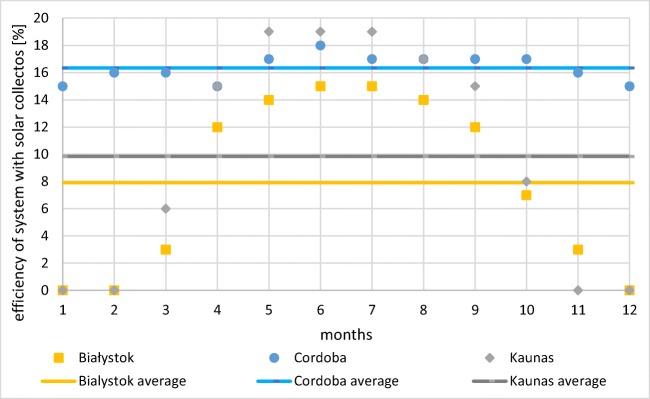
Mean total efficiency of the system (source: own elaboration)

Moreover, a mean monthly total efficiency of the system with an assumption that solar collectors are working continuously 24 hours a day was estimated (Fig. 13). The annual value in Spain was twice higher than in Poland (16% versus 8%), whereas in Lithuania, efficiency was slightly higher than in Poland (10%). Maximum monthly efficiency was found in Kaunas (19%). In both Poland and Lithuania, high variability was noted (15 and 19% respectively), opposite to Spain where the efficiency was nearly constant during the year (3% annual differentiation). It is worthy to note that monthly total mean efficiency of system was significantly lower than instantaneous values for collectors working in optimal solar radiation conditions, which were found by Krawczyk et al. (2018) at level 40–44%. This fact was connected with both mixing of cold and warm water in the tank, standing losses as well as methodology of the mean efficiency calculation, assuming 0 after sunset.

Even though the annual energy gathered from one collector in Cordoba (2030.62 kWh) was nearly equal to energy possible to obtain from the system with three collectors in Bialystok (2067.63 kWh) and two collectors in Kaunas (1904.32 kWh), it is worthy to note that a share of energy to be covered by solar system differed between months and locations significantly, as presented in Table 3.

**Table 3:** Percentage of covering energy needs for DHW by solar collectors in Bialystok, Cordoba, and Kaunas (source: own elaboration)

Month	Bialystok	Córdoba	Kaunas
	Percentage of covering energy needs for DHW by solar collectors [%]		
January	0.00	62.38	0.00
February	6.81	66.30	3.82
March	23.60	69.66	22.67
April	46.78	70.86	43.73
May	62.75	78.14	60.77
June	68.34	81.69	63.47
July	67.04	86.65	65.09
August	63.31	87.95	63.67
September	57.05	84.99	44.51
October	24.63	77.45	23.00
November	4.72	70.32	1.21
December	0.00	60.46	0.00

In Cordoba, one selected collector is the best solution, while the system requires the addition of energy supply. In the case of increasing number of collectors to two panels, it would appear the excess of solar energy share in an overall balance over the reference value (CTE 2017), laid down in context of overheating.

On the other hand, in Bialystok and Kaunas, similar solar gains but stronger warm water demand result in significantly lower share of solar energy in the overall balance, so the increase of solar panels could be considered.

Based on prices of energy (0.07 EUR/kWh for oil, 0.13 EUR/kWh for electricity) and an investment cost of main elements of the solar system (as well as labor costs relevant to them), simple payback time (SPBT) was estimated from Eq. (6):

6 $$ SPBT\left(\mathrm{years}\right)=\frac{N}{\varDelta Q} $$

where *N* is the investment cost in EUR, and Δ*Q* are the savings in EUR.

SPBT was calculated as 17–19 years. Taking into account any programs that support investments at level 60% (Spain) to 80% (Poland), the SPBT would be much shorter, just 4–8 years.

From an ecological point of view, modernization of the DHW systems in public buildings—replacing fossil fuels by solar collectors—would help to reduce the emission of pollutants. Considering, according to Report (CBES GUS 2015), a potential of 264.8 TJ of annual energy used in governmental buildings in Poland, energy supply as shown in Fig. 2 and assuming 80% covering these needs from solar systems would reduce CO_2_ emission by 17,248 tonne.

## Conclusions

In this paper, systems with solar collectors located on a roof with a tilt angle of 45° in Cordoba/Spain, whereas 35° in Bialystok and Kaunas, as recommended by Krawczyk et al. (2018), were analyzed. Efficiency of solar collectors was 3–4% higher in Cordoba than Bialystok and Kaunas. The results of this research lead to the following conclusions:

- The energy demand for DHW in Spanish office was significantly lower than in Poland and Lithuania as the result of higher network cold water temperature and national recommendation regarding low water usage per employee in office buildings.
- The energy gathered from the system with one collector in Cordoba was found to be three times higher than in Bialystok and twice higher than in Kaunas.
- In Spain, monthly gains were the highest in August, while in Poland in June and in Lithuania in May.
- Mean annual efficiency of the whole system was in all cases significantly lower (8–18%) comparing with momentary values of the collector efficiency (40–44%); however, both values were found the highest in Spain.
- The annual energy gathered from one collector in Cordoba was nearly equal to the energy for the system with three collectors in Bialystok and two collectors in Kaunas; however, in all cases, additional energy source is recommended to ensure proper temperature of water in winter, as well as a periodic disinfection.
